# First human surgery using a surgical assistance robotics device for laparoscopic cholecystectomies

**DOI:** 10.1007/s00464-023-10296-3

**Published:** 2023-08-21

**Authors:** Guy-Bernard Cadière, Jacques Himpens, Mathilde Poras, Luca Pau, Nicolas Boyer, Benjamin Cadière

**Affiliations:** 1 Digestive Surgery, Hôpital Universitaire St Pierre, ULB, Brussels, Belgium; 2 Digestive Surgery Institut Tzanck, Saint-Laurent-du-Var, France; 3 CHU St Pierre, Brussels, Belgium

**Keywords:** Laparoscopic robotics, Robotic-assisted cholecystectomy, First in human, Clinical trial, Minimally invasive surgical procedure, Robotic surgical procedures

## Abstract

**Background:**

Over the past 20 years, surgeons involved in soft tissue minimally invasive surgery have experienced the pros and cons of both conventional and tele-robotic laparoscopic approaches. The Maestro System, developed by Moon Surgical (Paris, France) aims to overcome the challenges inherent to both approaches thanks to a new concept that augments the surgeon’s performance at the bedside during a laparoscopic procedure.

**Methods:**

The current study aims to present the first human experience with laparoscopic cholecystectomy with the Maestro system on 10 patients.

**Results:**

All ten procedures were completed successfully. No significant complications related to the use of the Maestro system werenoted.

**Conclusion:**

Our preliminary observations appear to support the benefits of the Maestro system in non-emergent laparoscopic cholecystectomies. It goes without saying that further research is necessary to demonstrate the safety of this approach in other procedures.

Laparoscopic cholecystectomy (LCCE) is considered the gold standard for the surgical treatment of symptomatic gallstone disease [1, 2]. Cholecystectomy is one of the most commonly performed surgical procedures in the world. In the US approximately 700,000 LCCE surgeries are performed every year [3]. A major drawback lies in the fact that essential components of the surgical procedure must be delegated to surgical assistant(s) to manage vision (scope positioning) as well as access (tissue exposure). This distribution of roles and responsibilities creates limitations, as surgeons may encounter issues in controlling critical elements required for safety and efficiency throughout the procedure.

Mechanical support arms that hold the scope or ancillary instruments have been in use for some time. Mechanical arms ensure the stability of the scope image and allow appropriate exposure of the operative field. Unfortunately, manual locking and unlocking of scope holders representcumbersome workflow interruption for the surgeon. In addition, feedback-deprived mechanical retraction may lead to significant injury of fragile organs.

Tele-robotic systems specifically address the challenges created by the need to delegate essential parts of the laparoscopic procedure. To address these issues, control of the instruments and vision have been given back to the surgeon. For instance, with the da Vinci™ surgical system (Intuitive Surgical Inc., Mountain View, CA, USA), the surgeon has full control of the endoscope and three ancillary instruments with a foot pedal.

The use of robots has proven to be very beneficial in laparoscopic prostatectomy, allowing identical or even better outcomes than conventional laparoscopy [4]. As a consequence up to 85% of prostatectomies are nowadays performed robotically [4]. However, the use of robotic technology brings about new unexpected limitations, including adaptability and situational awareness, linked with the fact that the surgeon is confined in a console [5]. The physical isolation of the surgeon, who is no longer scrubbed and gowned, prevents direct control of the operative field and impairs accurate understanding between robot, patient, and operating room team. These limitations created the need for improved communication between the operating room team and the surgeon.ignificant design efforts have been integrated in the more recent tele-robotic systems. to address the described shortcomings As a logic consequence, however, the complexity of setup and positioning significantly increased. It is to deal with this unwanted situation that a new platform has been elaborated by Moon Surgical (Paris, France).

Following more than 30 feasibility cases on cadaveric models, and after formal approval of the local IRB, we performed the first human cases with the Maestro Platform. The current study was designed and executed to demonstrate the feasibility and safety of the Maestro device to enhance performance in elective laparoscopic cholecystectomy.

## Materials and methods

### Study device

**Fig. 1:**
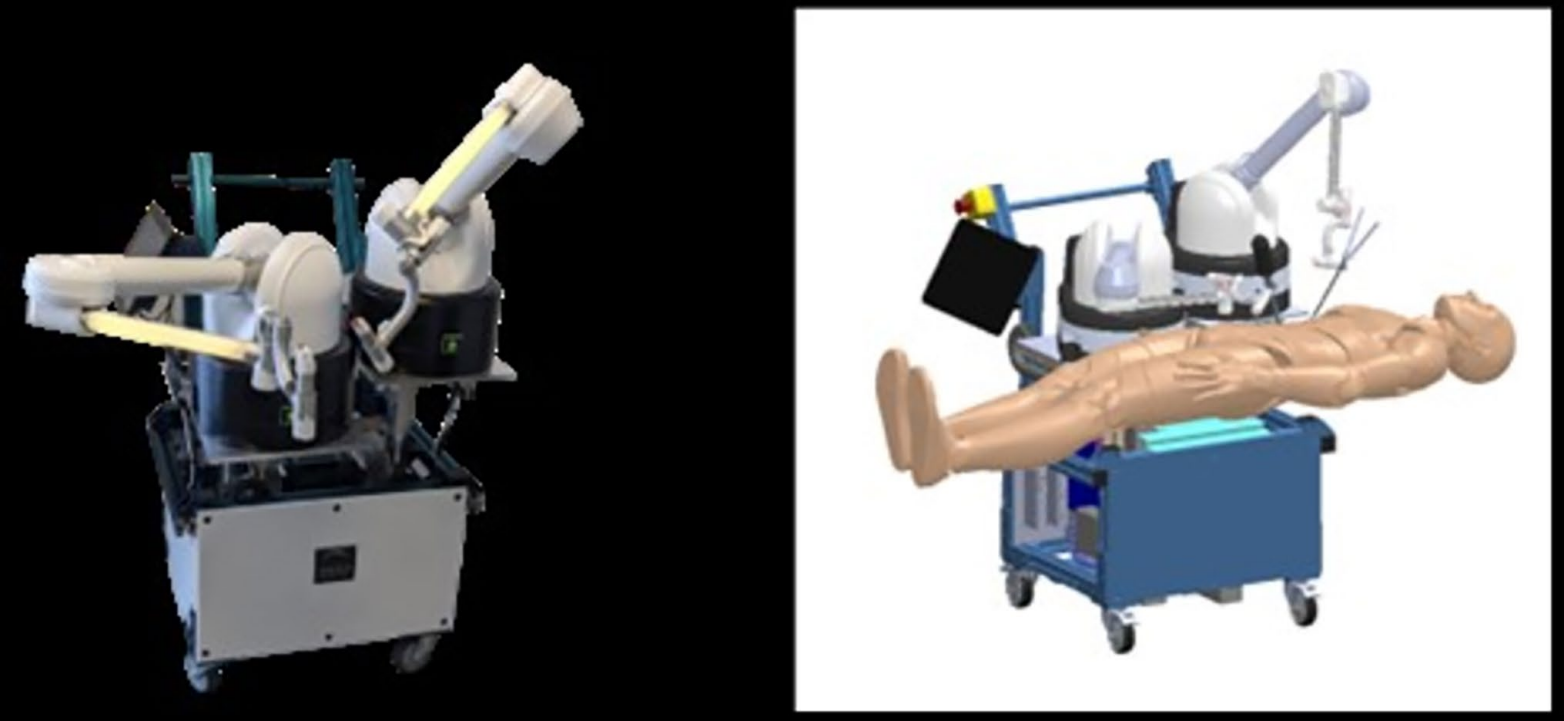
Schematic illustration of the Maestro Platform and its key touchpoint, and picture of the system in cholecystectomy position

The Maestro System (Moon Surgical SAS., Paris, France) is a two-arm platform device (Fig. 1) that holds and assists in the positioning of off-the-shelf laparoscopes and retractors during a laparoscopic intervention. Both arms of the Maestro platform have been designed to minimize mechanical friction and constantly compensate for the mass of the tool attached thanks to a proprietary software system.

The device allows for the instruments to be held statically or, alternatively, to be instantly repositioned by the surgeon. The instrument is automatically kept in place by the system whenever the surgeon releases the instrument. Repositioning the instrument is performed in complete mechanical transparency.

Each arm is mounted on a motorized independent platform which allows the user to position the system as required by the operative field and the patient configuration, which avoids arm clashes. Platforms can translate both on the vertical and horizontal axis. Color LED feedback indicators located on the arms provide the surgeon at all times with information about the state of each arm: idle, manipulating an instrument, or static holding.

### Study design and method

The First-in-Human study was designed as a single arm, prospective, single center, single operator and evaluator (GBC) feasibility trial. Ethics Committee and Competent Authority approvals for the clinical investigation were obtained from the FAMHP and an Ethics Committee designated by the College in accordance with the regulation (EU) 2017/745 on medical devices and in line with the law of December 22, 2020. The study was registered under: CIV-22-01-038769 (Eudamed), and NCT05243433 (clinicaltrials.gov ID) as per applicable regulations.

Adult patients scheduled for elective laparoscopic cholecystectomy were offered to participate freely. Patient screening and enrolment started in March 2022. Participants were provided with both oral and written information about the instrument and the current study and signed consent was obtained when the patients wereif willing to participate.

The primary objective of the study was to provide evidence for the feasibility of laparoscopic cholecystectomies using the Maestro Platform.

The primary safety endpoint was the safety of the procedure using the Maestro Platform without device-related serious adverse events at the time of procedure and within 30 days. Possible adverse events were e categorized as device or procedure related.

The primary effectiveness endpoint was the successful completion of the procedure using the Maestro Platform without conversion to a minimally invasive or open surgical procedure specifically due to a device failure or malfunction.

The demographic data included: age of the patient, gender, body mass index (BMI), previous abdominal surgery. Perioperative data included atypical anatomy, technical ease of the procedure independently from the system, surgeon’s comfort and satisfaction using the system, duration of the procedure and hospital stay.

When imaging data and laparoscopically observed data did not match, the patient was noted as having an atypical anatomy, butprecise description of the anatomy was obtained.

The surgeon’s comfort, satisfaction, and appreciation of the technical ease of the procedure were evaluated by the surgeon (GBC) immediately after the procedure. They were scored on a five-point scale. Incidence of device- and procedure-related AEs/SAEs during the hospitalization and one month after represented the primary safety endpoints. Adverse events were characterized using the Clavien-Dindo Classication (CDC).

The procedure duration time was determined as the time between the insertion of the scope and the time of placing the gallbladder in the extraction bag (Bagging).

### Surgical technique

**Fig. 2:**
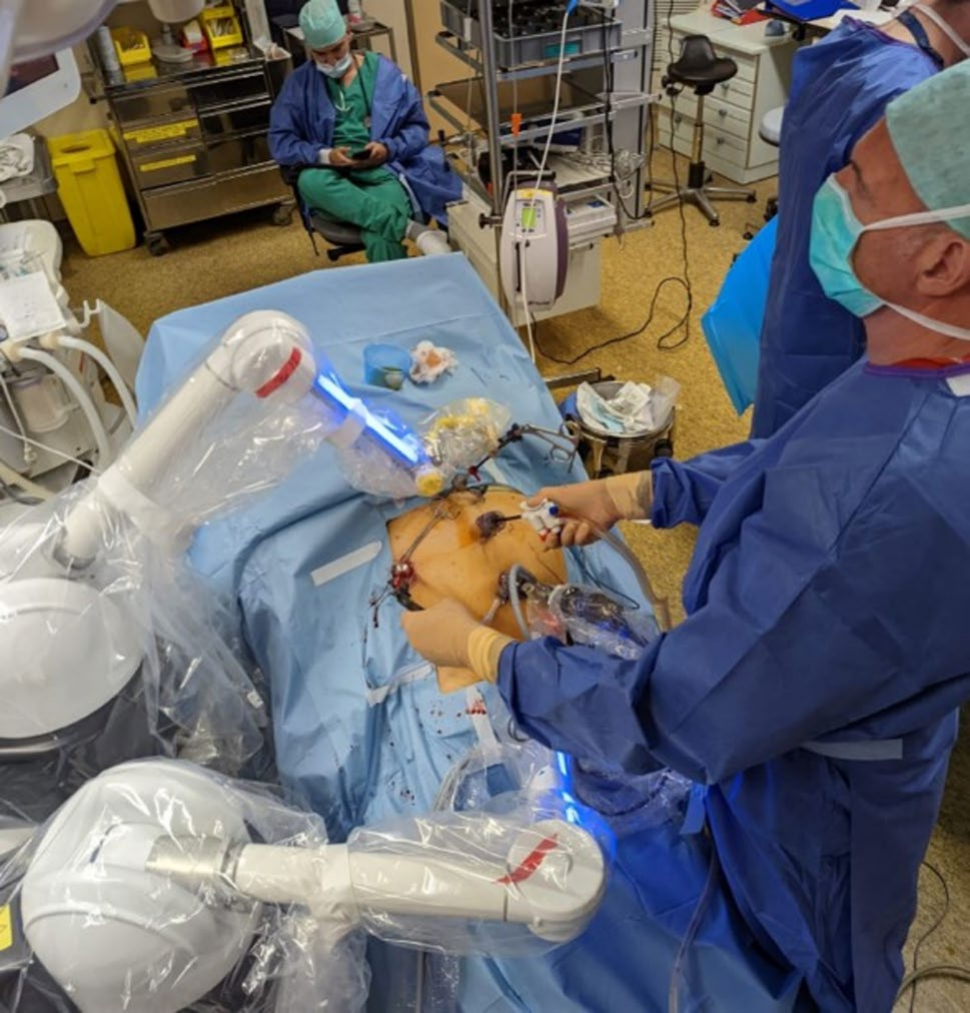
Disposition of the patient, the surgeon, the trocars, and the Maestro platform

The system was provided to the surgeon already draped and brought to the bedside after trocar placement and positioning of the patient in reversed Trendelenburg and slight lateral tilt on the left side of the patient. With our standard technique, cholecystectomy procedures were performed with the surgeon on the left side of the patient (Fig. 2).

Four trocars were used. A 5 mm epigastric port allowed the introduction of a grasping forceps (Fenestrated Grasping Forceps, Storz,) held by the Maestro system. It maintained constant traction on the liver unless adjustments were required for changes in visualization and exposure of the hepatocystic triangle (so called Calot’s triangle). A 10 mm trocar was inserted at the umbilicus for the 30-degree optical system held by the Maestro system. As per routine, the working trocars used by the surgeon wee two 5 mm ports, one placed in the right lateral subcostal position and one on the white line.

The grasping tool held by the surgeon’s left hand retracted the gallbladder to the right of the patient, in order to expose the Calot’s triangle and to obtain the critical view of safety, which is of the utmost importance because peroperative cholangiography are not commonly performed.

After dissection of the hepatocystic (Calot) triangle, and identification of the cystic artery and cystic duct, critical view of safety was achieved and both structures were clipped and divided without operative cholangiography. Dissection of the gallbladder from the liver bed was performed by retrograde technique. The gallbladder was then placed in a collecting bag and removed at the 10 mm umbilicus port site.

The surgeon operated in the “French” position. Thus, the surgeon’s left hand held the grasping forceps to manipulate the gallbladder. The surgeon’s right hand held the monopolar hook which was used to dissect. The left arm of the system was used to retract the liver, hence exposing the operating field, whilst the right arm of the system was holding the endoscope.

## Results

Of the ten participants who gave consent to participate in the study, all underwent the laparoscopic cholecystectomy using the Maestro System between April 13th and June 7th, 2022.

The patient demographics are summarized in Table 1. The mean age of the cohort was 59.5 years, most of the patients (70%) were females and the mean BMI (Body Mass Index) was 29.2 kg/m^2^.

**Table 1:** Patient demographics

Characteristic	Value
Total	10
Mean Age: years (range)	59.5 ± 18 (33–92)
Male sex: *n* (%)	3 (30%)
Mean BMI: kg/m^2^ (range)	29.2 ± 3.5 (21.8–34.6)

**Fig. 3:**
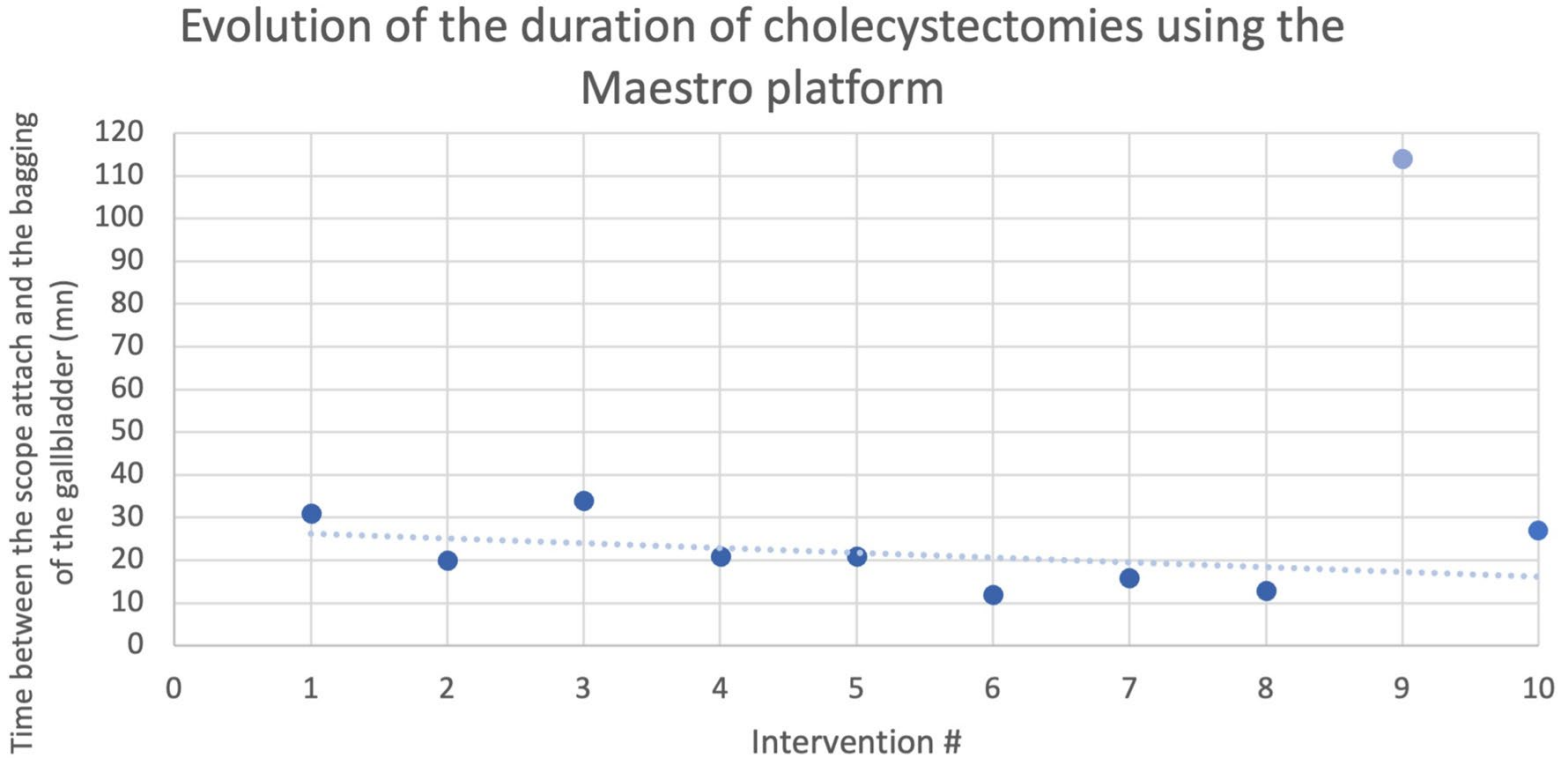
Duration of cholecystectomies using the Maestro platform

All 10 surgeries were successfully completed without the need to add a trocar, or to convert to a conventional minimally invasive, or open surgical procedure. No intraoperative complications related to the use of the Maestro System occurred. No external arm clashes were noted during any procedure. In 6 cases, the surgeon felt very satisfied. The surgeon felt satisfied in 3 procedures and mixed satisfied in 1 case. In the last seven cases, the surgeon felt very comfortable. The surgeon felt comfortable in 2 procedures and mixed in 1 case. In 6 cases, the technical ease of the procedure was evaluated as very easy, easy in 1 case, moderately easy in 2 procedures and difficult in 1 case. The anatomy was considered atypical in 1 case ( #9) only. The median duration of the surgeries was 21 min (Fig. 3). The procedural time showed a decrease [*x* = *k*(T10–T1)], NS with growing experience.

One retroperitoneal bleeding caused by a Veress needle puncture at initiation of the pneumoperitoneum required laparoscopic re-intervention the following day, and the patient stayed under surveillance for 19 days (Clavien-Dindo Grade IIIb). This was not related to the use of the Maestro system.

Upon initial examination of the insufflated abdomen, one of the patients (Patient #9) presented atypical anatomy: extensive adhesions in the abdominal cavity and a diseased gallbladder. The cystic duct could not be clipped and needed micro-suturing of the insertion site in the common bile duct with a 4.0 material resorbable suture. The intervention lasted 114 min. The technical use of the procedure was scored as “difficult” (4/5) by the surgeon, the comfort and the satisfaction levels were maximal.

The peroperative data are shown in Table 2.

**Table 2:** Peroperative data

Participant	Atypical Anatomy	Duration of the intervention (mn)	Peroperative complications	Clavien-Dindo Stage
1	No	31	None	
2	No	20	None	
3	No	34	Aortic puncture with the Veress needle	Grade IIIb
4	No	21	None	
5	No	21	None	
6	No	12	None	
7	No	16	None	
8	No	13	None	
9	Yes	114	None	
10	No	27	None	

The estimated blood loss was not registered for the 10 interventions, because it was considered as insignificant. One gallbladder was ruptured intra operatively (Case #9), which did not lead to any further complication. The average hospitalization time was 3.7 ± 5.40 days (range: 1–19). The 24 h post-operative pain levels were standard, with an average of 0.9 ± 1.5 (range: 0–4) and all patients were fully recovered at the 30 days follow-up call. The post-operative data are available in the Table 3.

**Table 3:** Postoperative data

Participant	24 h Post operative pain level (visual analog scale)	24H Pain level	30 days remission	Post-Op complication (< 30 days)	Duration of hospitalization
1	2	2	Confirmed	No	1
2	0	0	Confirmed	No	2
3	4	4	Confirmed	No	19
4	0	0	Confirmed	No	2
5	0	0	Confirmed	No	2
6	0	0	Confirmed	No	2
7	3	3	Confirmed	No	2
8	0	0	Confirmed	No	2
9	0	0	Confirmed	No	3
10	0	0	Confirmed	No	2

The retraction of the liver by the fenestrated grasping forceps held by the Maestro system did not lead to any liver perforation or injury.

The pathological anatomy of each gallbladder was then analyzed by the hospital’s pathology department.

The analyses are listed in Table 4. Seven gallbladders presented a discrete chronic cholecystitis, 2 gallbladders presented a chronic cholecystitis, and 1 gallbladder did not present any lesions.

**Table 4:** Gallbladder pathological anatomies

Participant	Gallbladder pathological anatomy
1	Discrete lithiasis chronic cholecystitis
2	Discrete lithiasis chronic cholecystitis
3	Discrete lithiasis chronic cholecystitis
4	Normal lithiasis gallbladder
5	Chronic lithiasis cholecystitis
6	Discrete lithiasis chronic cholecystitis
7	Discrete lithiasis chronic cholecystitis
8	Chronic lithiasis cholecystitis
9	Chronic lithiasis cholecystitis
10	Discrete lithiasis chronic cholecystitis

## Discussion

The rationale for the proposed Maestro LIFT-OFF study is to deal with the issue that in conventional laparoscopic procedures the surgeon is highly dependent on his/her assistant, for scope manipulation as well as operating field exposure through retraction. By overcoming this limitation, the Maestro Platform would allow surgeons to optimize their time and efficiency, without the limitations encountered with tele-robotic systems.

With the current embodiment. He surgeon was satisfied to very satisfied with the assistance provided in 9 cases out of 10. The surgeon felt neutral to the level of assistance provided in case #1 only. Critical point is probably that each arm of the Maestro Platform provides the surgeon a unique feeling of mechanical transparency. Even when a heavy laparoscope is attached, Maestro creates a sense of weightlessness, which benefits to positioning and creates haptic feedback. Although a recurrent issue with robotic assisted surgery [6], no arm clashes were observed during the procedures and the surgeon could manipulate the instruments without resistance, without feeling their mass, or the arm offering resistance. In the first case only did the surgeon not feel satisfied or very satisfied, which shows a steep adaptation (learning) curve.

Furthermore, the surgeon felt comfortable to very comfortable in 9 out of 10. Cases, and felt neutral regarding the comfort in case #1 only. The superior image stability gives the surgeon the possibility to look away from the screen and come back with the exact same image. This vision stability also allows for an increased accuracy in delicate tasks such as micro-suturing as experienced in patient #9. In this particular case the surgeon scored the comfort as maximal, although the patient had an atypical anatomy, the technical ease was considered as difficult, and the intervention was lengthy compared to the other cases.

Unlike the other robotic techniques, the Maestro device allows the surgeon to stay at the patient’s bedside without the need to constantly verbalize his/her orders as required by the distance to the patient. In case #9 the Maestro System showed adaptability to an unplanned anatomy. In the first case only, the surgeon did not feel comfortable or very comfortable, which again shows a steep adaptation curve.

Time was measured between the scope attachment and the bagging of the gallbladder to avoid the variation in extraction time linked to the state of the gallbladder (depending on the size and number of stones the extraction of the bag containing the gall bladder can be significantly more or less time consuming). The graph #1 suggests a swift learning curve, without however reaching statistical significance.

After only 1 case, the surgeon felt comfortable and satisfied with the assistance provided. Same as with the technique, instruments and the surgeon’s disposition were kept unchanged compared to traditional laparoscopic cholecystectomy. The system simply has to be approached to the bedside once the patient is in the correct position, and the trocars have been positioned.

Given the objective of the system (i.e., providing the surgeon with the best assistant, whilst keeping the same surgical practice), the learning curve was very steep, requiring only in vitro training on a synthetic model, in which LED feedback, instrument coupling and arm positioning were incorporated.

Our results on this new approach did not demonstrate an increase in postoperative complications compared to conventional laparoscopic cholecystectomy. Moreover, because the system integrated existing and well experienced laparoscopic techniques and operating room routine the surgeon obtained proficiency with minimal training.

We believe that the Maestro° device will actually be beneficial for young surgeons as well as trainees because it does not change anything to the surgical technique but simply facilitates the surgical manipulation. Moreover, during proctoring, it would allow the proctor surgeon to focus on guiding essential parts of the procedure without wasting attention or energy to unessential parts such as stability of the optical system or exposure of the operative field.

The primary safety and effectiveness endpoints were met in this series of 10 cholecystectomy patients, demonstrating the feasibility of laparoscopic assistance using the Maestro Platform.

Additional data on more patients and diverse clinical indications, such as hernias, and large operative fields including colectomies and bariatric procedures will obviously be needed to further confirm the safety of this approach. Last but not least, the financial aspect will be an important factor towards the spread of the system.

The Maestro platform provides the surgeon a stable vision and resistance free manipulation of all the surgical tools. Moreover, the surgeon can remain at the bedside, and keep in direct communication with the surgical team. In conclusion, thanks to the new platform the surgeon can be completely and at all times devoted to the intervention.
